# Analysis of minimum inhibitory concentrations of azithromycin, ceftriaxone and ciprofloxacin for *Neisseria gonorrhoeae* from antimicrobial resistance sentinel surveillance, Germany, 2014 to 2023

**DOI:** 10.2807/1560-7917.ES.2026.31.34.2500803

**Published:** 2026-08-27

**Authors:** Lena Böff, Kathleen Klaper, Achim Dörre, Annemarie Pantke, Susanne Buder, Sandra Dudareva, Viviane Bremer, Dagmar Heuer, Klaus Jansen, Regina Selb, the Go-Surv-AMR study group, Go-Surv-AMR study group, Josef Köstler, Michaela Simon, Susanne Polywka, Nicole Degel-Broßmann, Tobias Wienemann, Colin R MacKenzie, Ralf Ignatius, Crista Jebelean, Martin B Koeppel, Klaus-Peter Hunfeld, Daniela Walch, Matthias Marschal, Judith Overhoff, Andreas Wille, Heike Granzer, Werner Wenzel, Alexander Halfmann, Sören L Becker, Patrick Chhatwal, Galina Asseva, Kerstin Neumann, Martin Eisenblätter, Barbara Bauer, Henriette Hoffmeister, Michael Lefmann, Holger Rüssmann, Sabine Waldmann, Stefan Ziesing, Sebastian Grund, Maria Raptaki, Tilo Hackel, Annika Baugartz, Bianca Petermann, Roland Pfüller, Ana-Gabriela Sitaru, Petra Lüthje, Sabine Grazina Pociuli, Kirsten Hage, Heidrun Peltroche, Sabine Gröbner, Andreas Groß, Vanessa Dreyer, Ute Tonnemacher, Gerhard Haase, Helga Haefner, Veronika Balau, Daniela Nagel, Simone Korten, Regine Neumann, Bernd M Grüner, Martina Kerwat, Nadine Dietze-Jergus, Norman Lippmann, Meike Wedemeyer, Bettina Tiemer, Tobias Löwe, Babett Viete-Wünsche, Anna Trimborn, Marlis Gerigk, Lisa Marr, Jörg Steinmann, Neele Judith Froböse, Michael Probst-Kepper, Stefanie Witzke, Andi Krumbholz, Ekkehard Siegel, Moritz Brandstetter, Gregor Maschkowitz, Helmut Fickenscher, Carolin Muhl, Doris Streit-Schmid, Thomas Back, Arthur B Pranada, Martin Obermeier, Christina Pappa, Eva-Maria Moench, Imke Friedrichs, Valérie Le Saout-Chapot, Roman Schwarz, Anja Potthoff, Meike Grübmeyer, Marcus W Thomé, Maria Zimmermann, Anja Berger, Philip Eisermann, Rebecca Hinz

**Affiliations:** 1 Department for Infectious Disease Epidemiology, Robert Koch Institute, Berlin, Germany; 2 Postgraduate Training for Applied Epidemiology, Department for Infectious Disease Epidemiology, Robert Koch Institute, Berlin, Germany; 3 ECDC Fellowship Programme, Field Epidemiology path (EPIET), European Centre for Disease Prevention and Control (ECDC), Stockholm, Sweden; 4 Department for Infectious Diseases, Robert Koch Institute, Berlin, Germany; 5 Reference Laboratory for gonococci, Robert Koch Institute, Berlin, Germany; 6 The members of the Go-Surv-AMR study group are listed under Collaborators

**Keywords:** Minimum Inhibitory Concentration, Bacterial Sexually Transmitted Diseases, Neisseria gonorrhoeae Infection, Antimicrobial Drug Resistance, Sexual Health

## Abstract

**BACKGROUND:**

*Neisseria gonorrhoeae* (NG), the pathogen causing the sexually transmitted infection (STI) gonorrhoea, shows increasing antimicrobial resistance threatening recommended treatments.

**AIM:**

We examined temporal trends in antibiotic susceptibility of NG isolates in Germany and possible shifts in sexual networks.

**METHODS:**

We analysed annual data from the German antimicrobial resistance sentinel surveillance programme Go-Surv-AMR (2014–2023). Isolates were tested by E-test for phenotypic susceptibility to azithromycin (AZM), ceftriaxone (CRO) and ciprofloxacin (CIP) to determine minimum inhibitory concentrations (MIC). We described patient demographics and calculated MIC metrics by year, sex, age group and specimen type. We assessed differences in yearly MIC distributions using Kolmogorov-Smirnov tests and analysed temporal trends by fitting beta distributions to log-transformed MIC by antibiotic and epidemiological group.

**RESULTS:**

We collected 5,421 NG isolates from across Germany, primarily from males (n = 4,704) and from urethral specimens (n = 4,060). From 2016 to 2023, median AZM MIC nearly quadrupled in female and male isolates (0.12–0.45 mg/L). Median CRO MIC remained low overall (≤ 0.009 mg/L) but increased significantly in males in 2022–2023, especially in extragenital/screening isolates. Median CIP MIC increased (0.003–0.0057 mg/L), most pronounced in male extragenital/screening isolates.

**CONCLUSION:**

Across the examined decade, NG showed subtle increases in MIC in extragenital/screening isolates from males, possibly reflecting decreasing antibiotic susceptibility in groups that regularly undergo STI screening. Rising AZM MIC suggests early spread of resistant strains. This highlights the need for continued surveillance, consistent susceptibility testing, targeted education for vulnerable groups, promotion of condom use, and potential vaccination.

**Key public health message**

**What did you want to address in this study and why?**

Gonorrhoea is a sexually transmitted infection (STI) that is common among sexually active men who have sex with men. Antibiotic resistance in *Neisseria gonorrhoeae*, the bacterium causing gonorrhoea, can make it harder to treat the disease. We wanted to find out whether resistance to antibiotics in *Neisseria gonorrhoeae* has changed over the past 10 years in Germany and if some groups are more affected than others.

**What have we learnt from this study?**

We used data collected from laboratories across Germany in a national surveillance system for gonorrhoea to analyse the development of antibiotic resistance. We found that the bacterium is becoming less responsive to the three antibiotics that we examined. This was especially true for samples from males that were not collected from the genital region.

**What are the implications of your findings for public health?**

Our findings demonstrate the importance of monitoring antibiotic resistance in gonorrhoea. This requires laboratory testing when patients are diagnosed and can help doctors choose appropriate treatments. General STI prevention is important, particularly for men who have sex with men and young heterosexual persons. Easy access to STI testing, timely treatment, safer sex and future vaccination could all help to reduce the spread of gonorrhoea.

## Introduction

The ongoing acquisition of antimicrobial resistance (AMR) of *Neisseria gonorrhoeae* (NG) continues to pose a major public health threat, intensifying concerns of treatment failures and challenging the control of gonorrhoea globally [1-3]. In most countries, including Germany, the third generation cephalosporine ceftriaxone (CRO) is the last recommended option for empiric monotherapy [4-6]. In Germany, the national Gonococci-Resistance-Network (Go-Surv-AMR), a collaboration between the Robert Koch Institute (RKI) and over 60 primary laboratories nationwide, has monitored AMR in NG since 2014 [7]. For azithromycin (AZM), which is still recommended in Germany in combination with CRO in case the recommended follow-up visit for test of cure is uncertain, the fraction of isolates above the epidemiological cut-off value (ECOFF) has risen from below 1% in 2017 to over 25% in 2023 [8-10]. In contrast, resistance to CRO in NG has remained consistently low with fewer than 0.5% resistant isolates in Germany each year. In surveillance by Go-Surv-AMR, high resistance levels against ciprofloxacin (CIP) have been continuously measured [10]. However, CIP can still be administered if susceptibility is demonstrated [4].

Mandatory notification of all laboratory-confirmed gonorrhoea cases according to §7.3 of the German Infection Protection Act (IfSG) was introduced in 2022, but an electronic reporting system is not yet implemented. Thus, detailed epidemiological and clinical data on affected groups for gonorrhoea are not available for Germany [11]. However, the notification of NG with reduced susceptibility to AZM, cefixime and CRO is mandatory since 2020, allowing more detailed data collection for cases from then onwards.

Approximately half of all male gonorrhoea cases in Germany and in other European countries are men who have sex with men (MSM) [12-15]. Sexually transmitted infections (STI) are common among MSM. In Germany, fewer female (< 10%) than male gonorrhoea cases with reduced antibiotic susceptibility have been notified. Similarly, fewer NG isolates submitted via Go-Surv-AMR are from females. More recently, an increase in NG infections has been reported among young heterosexual adults, both males and females, in several countries [14,15]. Understanding possible resistance patterns within specific demographic or behavioural subgroups is crucial for targeted public health strategies.

Irrespective of clinically relevant resistance breakpoints, the minimum inhibitory concentration (MIC) allows susceptibility characterisation in detail. Subtle changes in NG MIC distributions can provide insights into evolving patterns and serve as early warning signals for future emerging resistance. We performed a detailed temporal MIC analysis of isolates submitted to Go-Surv-AMR between 2014 and 2023 for three important antibiotics. We assessed differences in MIC shifts and resistance developments by sex, across different age groups, settlement structure and by specimen type over time.

## Methods

### Dataset, antimicrobial susceptibility testing, variables

Laboratories within the Go-Surv-AMR network submit gonococcal isolates and basic epidemiological and clinical data to the RKI where the isolates are centrally retested for AMR. Isolates and epidemiological data submitted between 2014 and 2023 were included in the analysis. Data from cases notified to the RKI from 2020 onwards included additional information. The isolates were centrally cultured and tested for phenotypic susceptibility to AZM, CRO and CIP using E-tests to determine MIC, as described previously [7]. We used MIC values as read from the E-tests (doubling dilutions and half-dilutions). The MIC values for CIP were right-censored at 32 mg/L due to the limit of dilution steps. As precise MIC values were not available for all isolates between October and December 2023, this period was excluded from the analyses. The following demographic case data were considered for each isolate, whenever available: year of diagnosis (alternatively: year of centralised testing at the RKI), age, administrative sex (in the German civil status law, administrative sex has three categories: female, male and diverse) and the 3-digit postcode of the patient (alternatively: postcode of the doctor’s office or laboratory). Using the 3-digit postcodes, we categorised areas with ≥ 1 million inhabitants as metropolitan city, cities with < 1 million inhabitants as medium-sized city, except for areas with < 100,000 inhabitants which were categorised as countryside.

Age was categorised as < 25, 25–34, 35–44 and ≥ 45 years. The analysis included individuals aged ≥ 14 years. Due to the small numbers of isolates from patients with diverse sex, these were not included in the stratified analyses by sex. The specimen type was recorded as anus/rectum, cervix, conjunctiva, pharynx, urethra, multiple or other. For analysis of isolates collected from males, those from the anal/rectal or pharyngeal region or multiple sites were categorised as extragenital/screening. Commonly, a pooled sample (urethral, anal, pharyngeal) is taken during STI screening and, thus, the actual location(s) of infection is unknown. We compared these NG isolates with those from urethral specimens as surveillance data show that samples from heterosexual males are predominantly urethral, whereas those from MSM are from various sites including the urethra, rectum, pharynx or multiple locations [10]. Urethral isolates are also more likely collected from symptomatic infections, while extragenital infections often present as asymptomatic [16]. Analyses were performed on isolates with available information on the respective variables; therefore, the number of isolates incorporated in each separate analysis differs and is indicated with each presented result.

### Statistical analyses

We visualised spatial coverage of submitted isolates across Germany at the 3-digit postal code level. Demographic data were described with absolute and relative frequencies for categorical variables: minimum, maximum, range, and interquartile range (IQR) for continuous variables. We performed descriptive analyses of the MIC values across time for AZM, CIP and CRO, including quantiles and MIC90 as the concentration inhibiting 90% of isolates. The descriptive metrics are reported in accordance with the MIC dilution steps as provided by the E-test scale and set in context to the standards for resistance (R), susceptible (S) and susceptible at increased exposure (I) specific for each antibiotic–pathogen combination as defined by the European Committee on Antimicrobial Susceptibility Testing (EUCAST; version 14.0, January 2024) [2].

To assess year-to-year differences in MIC distributions, we performed pairwise comparisons of measured MIC values for each antibiotic agent using the non-parametric Kolmogorov-Smirnov test, yielding p_KS_ values. Statistical significance was set at 5%. To further examine the development of MIC distributions between 2014 and 2023, we fitted beta distributions defined on the interval (0, 1), whereas shape was determined by two positive parameters alpha (*α*) and beta (*β*). More details of the beta distribution and an illustration showing its characteristics are available in Supplementary Section 1 and in Supplementary Figure 1. We fitted beta distributions for AZM and CRO separately across years and demographic strata. To account for the empirically observed two-peaked shape of MIC distributions in CIP, we used a mixture beta distribution model with two components per year and demographic stratum with *α_1_*, *β_1_* and *α_2_, β_2_* as the shape parameters for the two beta distributions, and *p_1_*Є (0, 1) as the weight of the first component.

To ensure compatibility with the beta distribution, we log-transformed the measured MIC values and rescaled them based on predefined MIC bounds to the interval (0, 1). We estimated shape parameters for each beta distribution via maximum likelihood estimation. The weight of the mixture components in the CIP models was also estimated for each year and across demographic strata. To visually evaluate the goodness of fit, we plotted and compared the empirical cumulative distribution functions (ECDFs) of the transformed and scaled MIC data to the theoretical values derived from the fitted (mixture) beta distributions, respectively. Furthermore, the fitted distributions were used to calculate distribution characteristics (mean, median, variance, 25% and 75% quantiles) on the original MIC scale for interpretation. This modelling approach was also applied in different strata to identify demographic factors with specific susceptibility patterns. The statistical analyses were conducted using R in version 4.1.3 (https://www.r-project.org/) and R Studio (http://www.posit.co) with the packages ggplot2 for visualisations [17] and sf for geospatial mapping [18] with a shapefile indicating 3-digit postal code areas (https://welcome.openstreetmap.org/what-is-openstreetmap/).

## Results

### Temporal and spatial coverage

**Figure 1:**
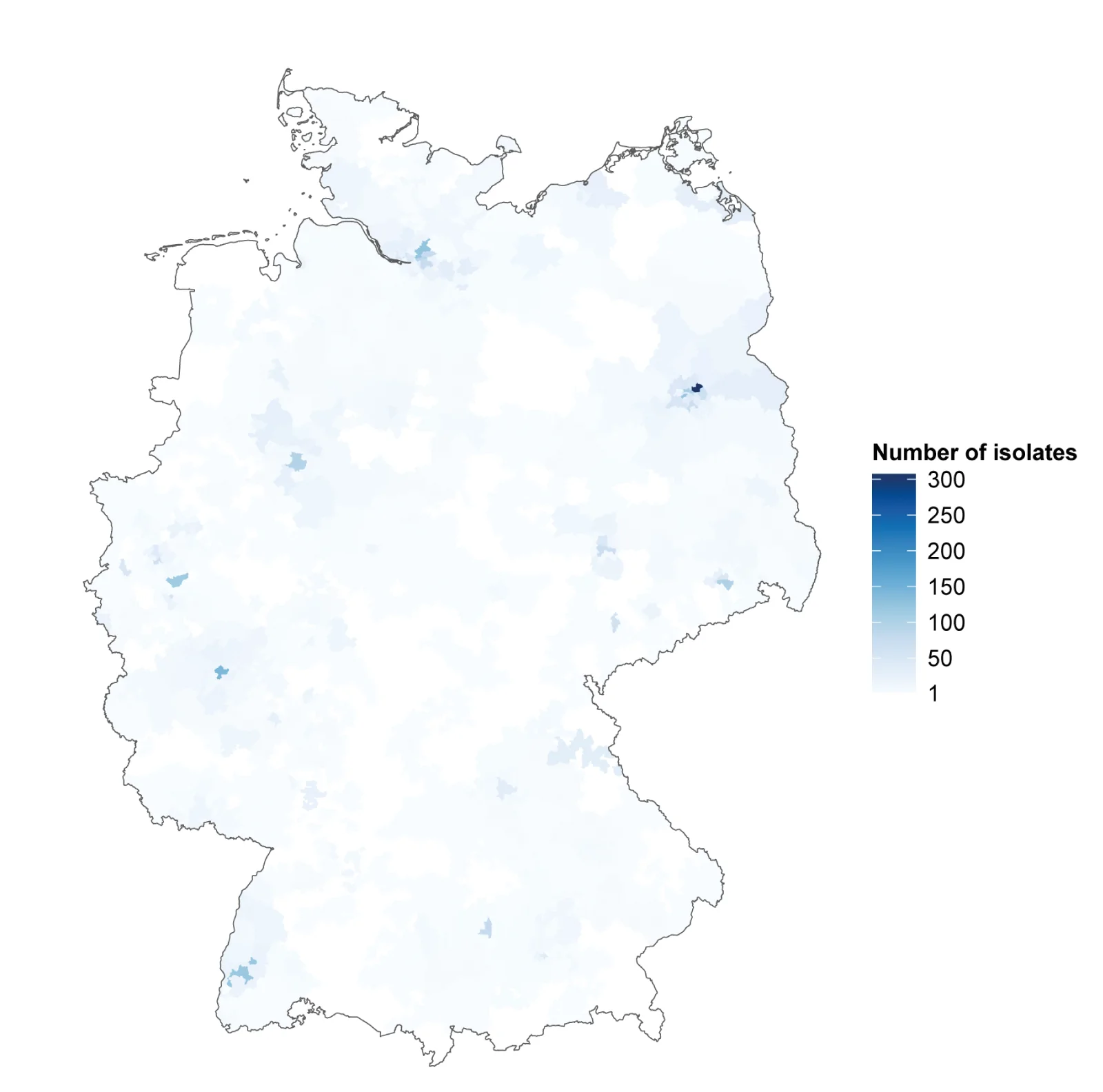
Geographical distribution of cases with *Neisseria gonorrhoeae* isolates from sentinel surveillance, Germany, 2014–2023 (n = 5,421)^a^

^a^ Geographical location was based on the 3-digit postcode of the residence of the patient (alternatively: postcode of the doctor’s office or laboratory).

In the analyses, we included 5,421 viable NG isolates from 2014 to 2023, with increasing case numbers recorded over the years (Table 1). The isolates were sent to the RKI by 114 laboratories across all 16 federal states in Germany with higher numbers of isolates from more densely inhabited areas (Figure 1). The highest case numbers were from Berlin (n = 1,411; 26.0%), North Rhine-Westphalia (n = 897; 16.5%) and Hamburg (n = 573; 10.6%).

**Table 1:** Characteristics of female and male cases with *Neisseria gonorrhoeae* isolates from sentinel surveillance, Germany, 2014–2023 (n = 5,295)^a^

Characteristics	Females		Males	
	n	%	n	%
All	591	11.2	4,704	88.8
Year				
2014	29	4.9	281	6.0
2015	34	5.8	316	6.7
2016	52	8.8	460	9.8
2017	61	10.3	452	9.6
2018	33	5.6	387	8.2
2019	60	10.2	404	8.6
2020	53	9.0	458	9.7
2021	46	7.8	358	7.6
2022	118	20.0	782	16.6
2023	105	17.8	806	17.1
Age (years)				
Mean	35		36	
Median	30		33	
Range	14–82		14–87	
IQR	23–43.75		26–44	
Age group (years)				
< 25	185	31.3	907	19.3
25–34	166	28.1	1,656	35.3
35–44	98	16.6	1,003	21.3
≥ 45	141	23.9	1,122	23.9
Missing	1	0.2	16	0.3
Specimen type				
Anus/rectus	5	0.8	301	6.4
Cervix	413	69.9	NA	
Conjunctiva	8	1.4	13	0.3
Multiple	2	0.3	16	0.3
Other	17	2.9	22	0.5
Pharynx	6	1.0	38	0.8
Urethra	59	10.0	4,001	85.1
Missing	81	13.7	313	6.7
Settlement^b^				
Countryside	167	28.3	1,428	30.4
Medium-sized city	172	29.1	1,327	28.2
Metropolitan city	252	42.6	1,949	41.4

IQR: interquartile range; NA: not applicable.

^a^ In total, we received isolates from 5,421 cases. Information on sex was missing from 121 cases and 5 had diverse sex.

^b^ Settlement based on the 3-digit postcode of the address of the case. If missing, the 3-digit postcode of the doctor’s office or laboratory was used. Countryside: areas:  < 100,000 inhabitants;  medium-sized city: < 1 million inhabitants; metropolitan city: ≥ 1 million inhabitants.

### Demographics

Of the 5,421 isolates, 4,704 (86.8%) were from males,  591 (10.9%) from females and 5 (0.1%) from persons with diverse sex (Table 1). Information on sex was missing for 121 (2.2%) isolates. The median age in the overall study population and in males was 33 years, and 30 years in females. Overall and among males, the largest age group were 25–34-year-olds, among females those under 25 years. Most isolates were from urethral specimens overall (n = 4,109; 75.8.%) and from males (n = 4,001; 85.1%), while cervical isolates were predominant in specimens from females (n = 413; 69.9%). Most individuals resided in metropolitan cities (n = 2,254; 41.6%). Among males, the proportions of extragenital/screening isolates were higher in urban areas (medium-sized cities: n = 128; 9.6% and metropolitan cities: n = 172; 8.8%) than in rural areas (n = 52; 3.7%).

### Minimum inhibitory concentrations

The MIC values across the study period showed distinct trends for AZM, CRO and CIP (Table 2). In all three antibiotic agents, the measured MIC values were higher in 2014 and 2015 before decreasing to the lowest value in 2016.

**Table 2:** Determination of minimum inhibitory concentrations of azithromycin, ceftriaxone and ciprofloxacin for *Neisseria gonorrhoeae* isolates from sentinel surveillance, Germany, 2014–2023 (n = 5,421)^a^

Antibiotic	Quantile				KS results^b^	
	25%	50%	75%	90%	Compared with year	p value
Azithromycin						
2014	0.125	0.25	0.5	0.75	2015	0.0012
2015	0.094	0.19	0.38	0.75	2016	0.0000
2016	0.064	0.125	0.25	0.38	2017	0.3005
2017	0.094	0.125	0.19	0.38	2018	0.0345
2018	0.094	0.19	0.25	0.5	2019	0.0002
2019	0.125	0.19	0.38	1	2020	0.0682
2020	0.125	0.19	0.38	1.5	2021	0.0088
2021	0.125	0.25	1	1.5	2022	0.0485
2022	0.19	0.38	1	1.5	2023	0.0011
2023	0.19	0.5	1.5	1.5	NA	
Ceftriaxone^c^						
2014	0.003	0.012	0.032	0.047	2015	0.0000
2015	0.002	0.004	0.016	0.023	2016	0.0000
2016	0.002	0.003	0.012	0.023	2017	0.9999
2017	0.002	0.003	0.012	0.023	2018	0.8851
2018	0.002	0.003	0.008	0.023	2019	0.0017
2019	0.002	0.004	0.008	0.023	2020	0.9480
2020	0.002	0.004	0.008	0.016	2021	0.3635
2021	0.002	0.004	0.008	0.016	2022	0.8026
2022	0.003	0.004	0.008	0.012	2023	0.0000
2023	0.003	0.006	0.008	0.016	NA	
Ciprofloxacin						
2014	0.006	16	32	32	2015	0.0000
2015	0.003	2	16	32	2016	0.0110
2016	0.003	1.5	8	32	2017	0.0041
2017	0.003	2	12	32	2018	0.0091
2018	0.004	1.5	8	32	2019	0.0677
2019	0.004	3	8	32	2020	0.1051
2020	0.004	1.5	8	32	2021	0.0451
2021	0.006	3	8	16	2022	0.5140
2022	0.008	3	8	24	2023	0.0000
2023	0.008	6	16	32	NA	

KS: Kolmogorov-Smirnov test; MIC: minimum inhibitory concentration; NA: not applicable.

^a^ The MIC values were determined with E-test.

^b^ Kolmogorov-Smirnov test results present comparisons of measured MIC distributions between 2 years.

^c^ Number of isolates included:  5,419.

For AZM, statistical comparison by Kolmogorov-Smirnov tests between years showed significant differences in measured MIC distributions, except for between 2016 and 2017, and 2019 and 2020. The median MIC increased from 0.125 mg/L in 2016 and 2017 to 0.5 mg/L in 2023. Also, MIC90 reached the epidemiological cut-off of 1 mg/L in 2019, exceeding to 1.5 mg/L in all following years.

For CRO, MIC90 stayed below the clinical breakpoint (R > 0.125 mg/L) throughout the study period. After the median MIC values plateaued at 0.003–0.004 mg/L from 2015 to 2022, we observed an increase to 0.006 mg/L in the most recent year (2023). Six isolates in 4 years (one in 2015, 2018 and 2022, and three in 2023) from urethral specimens from males had MIC values above the clinical cut-off for CRO.

For CIP, MIC50 exceeded the clinical resistance cut-off (R > 0.064 mg/L) throughout the entire study period. From 2015 to 2022, MIC50 values ranged between 1.5 mg/L and 3 mg/L, before increasing to 6 mg/L in 2023. The MIC development over the study period is further detailed in Supplementary Figure 2.

### Beta distributions

The plotted ECDFs of transformed MIC data closely followed the estimated beta distributions as presented in Supplementary Figure 3 A–C.

**Figure 2:**
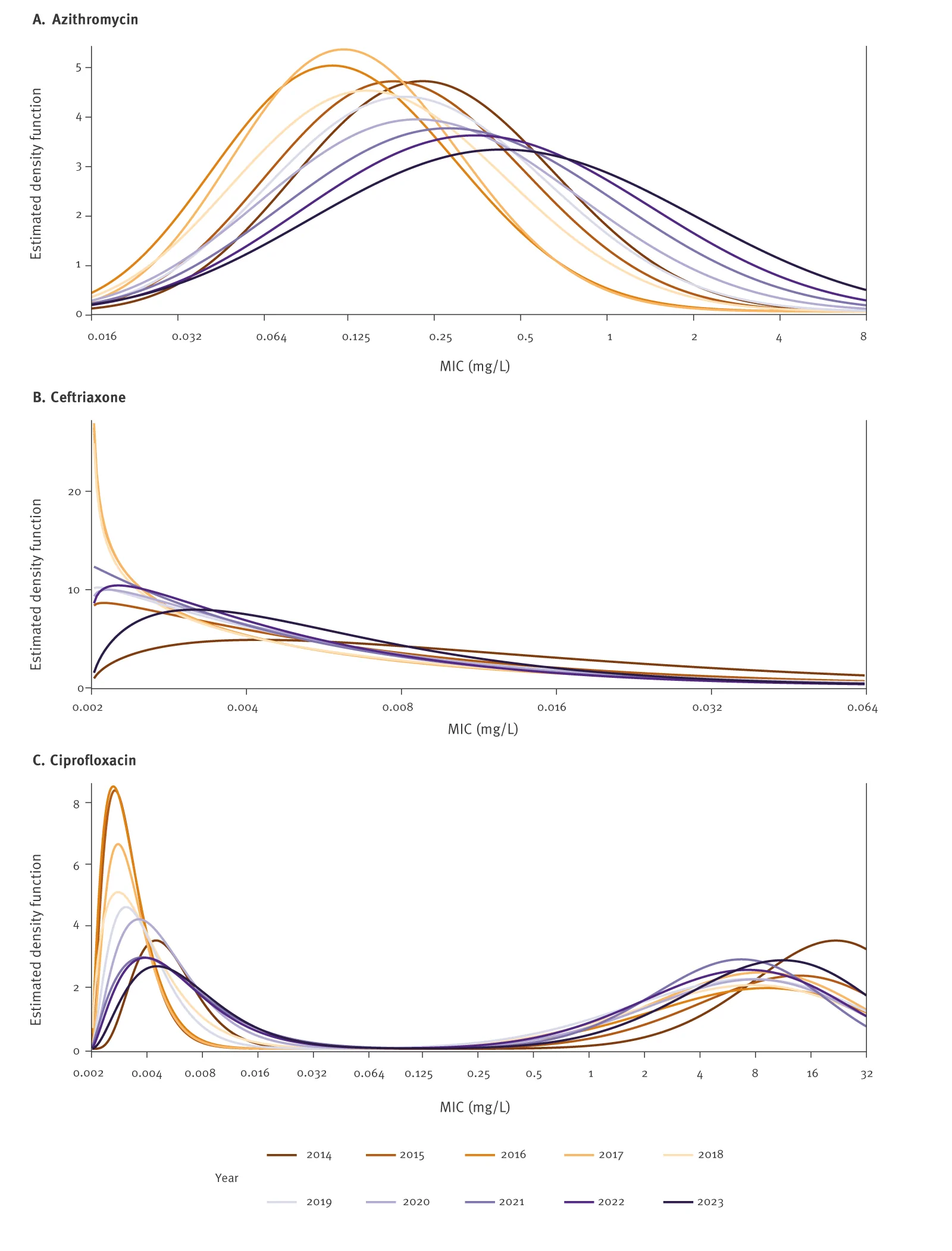
Estimated distribution of minimum inhibitory concentrations of azithromycin, ceftriaxone and ciprofloxacin for *Neisseria gonorrhoeae* isolates from sentinel surveillance, Germany, 2014–2023 (n = 5,421)^a, b^

After a minimum of 0.12 mg/L in 2016, the median MIC in AZM increased almost fourfold to 0.45 mg/L in 2023 (Figure 2). More details are presented in Supplementary Table 1. The 25% and 75% quantiles increased, with the upper increasing by more than five times between 2017 and 2023. Thus, the entire distribution moved upwards as displayed in Figure 2A showing the estimated distribution of the back-transformed MIC values across the years.

MIC: minimum inhibitory concentration.

^a^ Measured MIC values were first log-transformed to ensure compatibility of the multiplicative dilution scale with the beta distribution and then rescaled to the interval (0,1). Subsequently, shape parameters for each beta distribution were estimated via maximum likelihood estimation and fitted values were back transformed to the original MIC scale.

^b^ The MIC values were determined with E-test.

Panel B. For ceftriaxone,  5,419 isolates were included.

**Figure 3:**
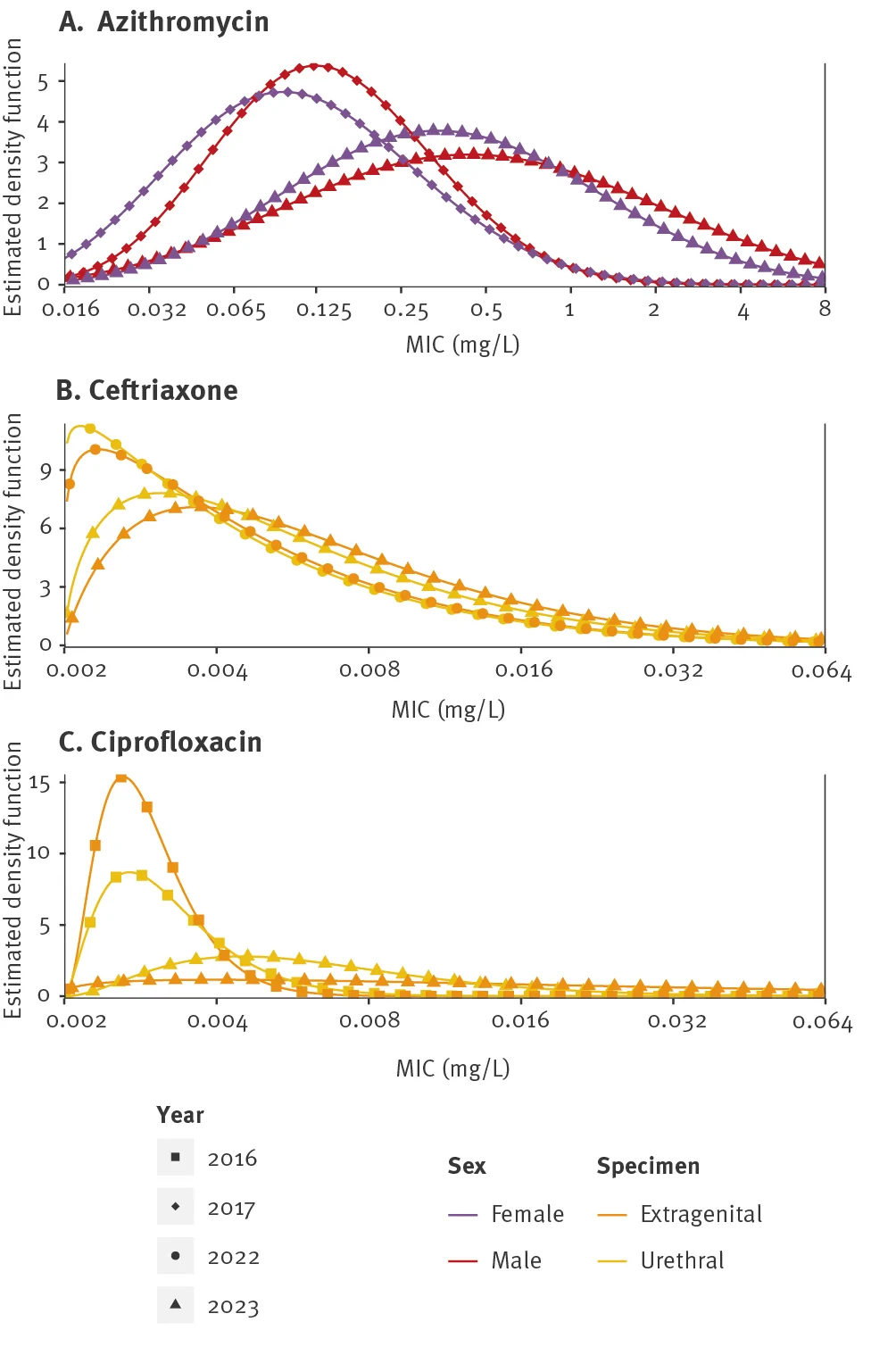
Estimated distribution of minimum inhibitory concentrations of azithromycin (n = 5,295), ceftriaxone (n = 4,354) and ciprofloxacin (n = 4,356) for *Neisseria gonorrhoeae* isolates from sentinel surveillance, by sex and specimen type, Germany, 2014–2023^a, b^

The increase in the median MIC was less pronounced in females than in males (females: 0.10 to 0.36 mg/L, p_KS_ < 0.01; males: 0.14 mg/L in 2017 to 0.46 mg/L in 2023, p_KS_ < 0.01) (Figure 3A). We were particularly interested in the development from 2017 to 2018, as a novel AZM resistance conferring locus was identified in 2018 [19]. From 2017 to 2018 and 2018 to 2019, median AZM MIC increased in males and females, but the difference in measured MIC distribution was only statistically significant from 2018 to 2019 (males: p_KS_: < 0.01; females: p_KS_: < 0.05), as presented in Supplementary Table 2. The early increase in AZM MIC was observed in all age groups except for the age group of 35–44 years where it was delayed by 1 year (2017: 0.15 mg/L; 2018: 0.13 mg/L; 2019: 0.23 mg/L; p_KS_ < 0.01 for 2018 to 2019), as shown in Supplementary Table 3. Isolates from all settlement categories and urethral and extragenital isolates showed an increase in median AZM MIC, as presented in Supplementary Tables 4–5. However, extragenital/screening isolates showed higher yearly median and quartile levels throughout the entire study period compared with urethral isolates.

MIC: minimum inhibitory concentration.

^a^ Measured MIC values were first log-transformed to ensure compatibility of the multiplicative dilution scale with the beta distribution and then rescaled to the interval (0,1). Subsequently, shape parameters for each beta distribution were estimated via maximum likelihood estimation and fitted values were back transformed to the original MIC scale.

^b^ The MIC values were determined with E-test.

Median MIC of CRO were low-level susceptible in all years (≤ 0.009 mg/L) (Figure 2B). More details can be seen in Supplementary Table 1. The median MIC values first decreased in 2015 and then remained relatively stable, fluctuating around 0.004 mg/L until 2022. We observed an increase in the median MIC from 0.0039 to 0.0048 mg/L in 2022–2023, accompanied by a statistically significant difference in the measured MIC distributions in the 2 years (p_KS_ < 0.001). This increase (p_KS_ < 0.001) was observed in the isolates from males (0.0038–0.0049 mg/L) but not in the isolates from females (0.0049 mg/L in both years), as presented in Supplementary Table 6. The increase was observed in all three settlement categories and all age groups, as presented in Supplementary Tables 7–8. Among males, the increase was more pronounced in extragenital/screening isolates (0.0039–0.0055 mg/L, p_KS_ = 0.01) than in urethral isolates (0.0037–0.0048 mg/L, p_KS_ < 0.001) (Figure 3B).

The estimated beta distributions for CIP revealed a clear bimodal structure in MIC values with the first component covering lower levels of the MIC scale (Figure 2C). More details are presented in Supplementary Table 9. The weight off the first component of the distribution decreased from 43% in 2015 to 32% in 2023, while the second component gained weight, therefore the distribution developed towards higher MIC values. The median MIC of the first component increased from 0.0030 mg/L in 2016 to 0.0057 mg/L in 2023. This shift in the lower part of the MIC values was visible among males (0.0030–0.0058 mg/L) and females (0.0030–0.0051 mg/L), as presented in Supplementary Table 10. Looking at different substrata in males, we found that this increase was slightly more pronounced in extragenital/screening isolates (0.0028 mg/L in 2016 to 0.0115 mg/L in 2023) than in urethral isolates (0.0030–0.0056 mg/L) (Figure 3C). Here the total raw MIC distributions of 2016 and 2023 were significantly different in both strata (p_KS_ < 0.001). Isolates from metropolitan cities reached slightly higher median MIC values (0.0061 mg/L) in 2023 compared with the countryside (0.0055 mg/L) and medium-sized cities (0.0053 mg/L), as shown in Supplementary Table 11. The second component covering upper levels of the MIC scale showed an initial decrease and plateaued at values slightly above 6 mg/L from 2019 onwards, before increasing to 9.5 mg/L in 2023 (Figure 2C). More information is presented in Supplementary Table 9. Differences in raw MIC distributions in 2022 and 2023 were statistically significantly different (p_KS_ < 0.001 for 2022/2023). This increase in the last year of the investigation was visible across all strata. It was particularly pronounced in medium-sized cities with a median MIC increase from 5.36 to 10.06 mg/L from 2022 to 2023 (p_KS_ = 0.017) and metropolitan cities (6.42–9.34 mg/L, p_KS_ = 0.001), whereas less evident in the countryside (7.03–9.64 mg/L, p_KS_ = 0.056), as presented in Supplementary Table 11.

## Discussion

In this study, we examined trends in MIC data for NG isolates collected within the German Go-Surv-AMR sentinel surveillance system between 2014 and 2023, presenting a decade-long investigation of MIC values for three clinically important antibiotics. By modelling MIC distributions using beta distributions, we were able to capture subtle changes of MIC values below the resistance threshold which might precede clinically relevant resistance.

Notably, we observed a significant shift to higher CRO MIC values from 2022 to 2023 at a level below the resistance threshold in isolates from males which was more pronounced in extragenital/screening than in urethral isolates. The MIC values for CIP shifted to higher levels between 2016 and 2023, especially for isolates below the resistance threshold in both females and males while this was again particularly visible in extragenital/screening compared with urethral isolates from males. Recent Go-Surv-AMR data show that extragenital/screening specimens predominantly originate from MSM in Germany, aligning with evidence that STI screening is primarily frequented by these persons [10,20,21]. We also observed a higher share of extragenital/screening isolates in metropolitan and medium-sized cities in our study. Data from the German microcensus indicated that 50% of lesbian, gay and bisexual persons reside in cities with over 100,000 inhabitants compared with one third of heterosexual persons [20,22]. The observed shift in CRO and CIP could therefore point especially to sexually active MSM with multiple sexual partners who might make use of regular STI screening offers. In general, CRO MIC distributions have remained relatively stable across Europe, with a sporadic occurrence of resistant isolates in some countries [23]. Hence, the recently observed rise in CRO MIC at the currently low levels and the shifts in CIP MIC over several years could be early indicators of further AMR development, potentially indicating a possible dynamic within MSM networks. As urethral specimens were presumably collected from a mixed population of mainly symptomatic patients of both MSM and heterosexual males, this heterogeneity could also explain the less pronounced but still observed CRO and CIP increase in these samples.

Due to recent reports by the European Centre for Disease Prevention and Control (ECDC) indicating a rise in NG notifications among young heterosexual adults [15], we were particularly interested to see age-specific trends in resistance developments. However, we did not observe substantial differences between age groups. This may indicate that sexual networks are relatively age-diverse or that the available epidemiological data was not sufficient to distinguish between these groups.

We wanted to explore if the previously observed increase in AZM resistance due to the mosaic *mtr* locus [19,24] is linked to certain epidemiological subpopulations. To explore this in detail, we examined MIC distributions particularly for developments from 2017 to 2018/19. Interestingly, despite the relatively small number of isolates from females in our study, the shift also appeared among those at an early stage. Our findings align with analyses based on data from the Euro-GASP network, which identified associations in AZM resistance with both females and MSM in 2016–2019 [23].

This early presence of elevated MIC values among females could either represent a direct introduction of resistant strains into the heterosexual population or a pre-existing circulation of strains containing the *mtr* mosaic at a low level. However, analyses of the clade harbouring the *mtr* mosaic locus in Go-Surv-AMR isolates from 2018 included only one female [19]. Therefore, the early shift of AZM in isolates from females might be independent of the *mtr* mosaic. In the United States, the *mtr* mosaic locus emerged as early as 2016 and was described predominantly in MSM [25].

The small sample size of isolates from females in our study population might reflect several aspects. Gonorrhoea is often asymptomatic in females and therefore less frequently diagnosed. Additionally, vaginal swabs are a common specimen type in females and useful for nucleic acid amplification testing (NAAT). Culture for NG is often not successful from vaginal swabs due to low bacterial load and possible interference from commensal flora [26,27]. Instead, endocervical swabs, which are more appropriate for culturing, are less frequently collected. Furthermore, female partners of symptomatic cases are frequently treated without being tested. Hence, it is plausible that a higher share of isolates from females included in our study population were tested for susceptibility after treatment failure, possibly due to AZM resistance. In Germany, azithromycin is still used for treatment of STIs before the confirmation of the causative pathogen [7,9]. This may have caused a bias in our sample with overrepresentation of resistant isolates from females.

However, some limitations should be noted. Currently, no notification data on the incidence or prevalence of NG in Germany are available which could help guiding sample selection. Even though the number of laboratories participating in the laboratory network has increased throughout the years, the sentinel data do not include a comprehensive collection of all NG samples in the German population and might therefore not be fully representative.

In the E-test we used, the highest concentration of CIP was MIC 32 mg/L which sets a limit for the beta distribution to accurately model the shape of the distribution near the tail on the right side. Thus, we could not estimate the proportion of isolates with MIC values considerable higher than 32 mg/L, thus biasing the MIC distributions and estimates for CIP.

Nonetheless, we used a large nationwide dataset from the German sentinel surveillance system to apply beta distribution modelling of MIC flexibly in a stratified analysis. This provided statistically derived effect measures for all three investigated clinically relevant antibiotic agents and a higher sensitivity to detect MIC shifts even within susceptible ranges. Our current findings may point towards developments in possibly vulnerable population groups and may serve as early warning signals for evolving resistance patterns. With CRO currently remaining the last reliably effective antibiotic agent recommended for empiric monotherapy, the rising MIC trend is concerning, even if currently observed at a low level. Therefore, continuous and intensified resistance surveillance in NG remains crucial to inform treatment guidelines promptly. In addition, special emphasis should be given to tailored public health recommendations highlighting the importance of prevention to avoid STI in general. Targeted STI screening offered especially for MSM is an important measure to capture subclinical NG infections in an early stage. However, regular STI screening of asymptomatic individuals, e.g. in users of HIV pre-exposure prophylaxis, can lead to increased antibiotic consumption and may also result in selective pressure of strains with increased resistance [28]. Doxycycline post-exposure prophylaxis (Doxy-PEP) may pose a risk of further antimicrobial resistance developments in antibiotics other than doxycycline [29-31]. The high tetracycline resistance observed in Go-Surv-AMR and Europe suggests limited effectiveness of Doxy-PEP for gonorrhoea prevention [30].

## Conclusion

Surveillance of antibiotic resistance in NG is a crucial tool to provide early warning signals on AMR developments. Emphasis should be placed on consistent susceptibility testing of NG in primary diagnostics and promotion of further preventive measures focusing on most affected groups. These include the promotion of continued condom use and potentially vaccination against NG.

## Data Availability

Data supporting the findings of this study are available from the corresponding author upon reasonable request.

## Supplementary Data

698000 Supplementary Material
