# Supplementary material for: Analysis of minimum inhibitory concentrations of azithromycin, ceftriaxone and ciprofloxacin for Neisseria gonorrhoeae from antimicrobial resistance sentinel surveillance, Germany, 2014 to 2023
Source: Euro Surveill. 2026 Aug 27;31(34):2500803. doi: 10.2807/1560-7917.ES.2026.31.34.2500803 (PMC13525358; doi:10.2807/1560-7917.ES.2026.31.34.2500803)
Supplement: Supplementary file 1 [file 25-00803_BOEFF_Supplement.pdf]

Supplement

This supplementary material is hosted by Eurosurveillance as supporting information alongside the article "Decreased antibiotic susceptibility in extragenital and urethral isolates of Neisseria gonorrhoeae in men: An analysis of minimum inhibitory concentrations from Germany's antimicrobial resistance sentinel surveillance, 2014–2023", on behalf of the authors, who remain responsible for the accuracy and appropriateness of the content. The same standards for ethics, copyright, attributions and permissions as for the article apply. Supplements are not edited by Eurosurveillance and the journal is not responsible for the maintenance of any links or email addresses provided therein.

Supplementary Section 1

The beta distribution is a flexible continuous probability distribution defined on the interval (0,1). It is determined by two positive parameters, α and β, which control the shape of the distribution. Depending on these parameter values, the beta distribution can represent a wide range of patterns, including approximately uniform, symmetric, left-skewed, right-skewed, or more concentrated distributions. This flexibility makes it useful as a parsimonious two-parameter model for bounded data.

In our analysis, the beta distribution was not applied to the raw MIC values directly. Instead, MIC values were first log-transformed, which is appropriate because MIC measurements are obtained on a multiplicative dilution scale, and then rescaled to the interval (0,1). The beta distribution was subsequently fitted to these transformed values. This allowed us to summarize the overall shape of the empirical MIC distribution for each antibiotic using only two parameters, capturing not only central tendency but also broader features such as dispersion and skewness.

The beta density function is given by

$$f(x;\alpha,\beta) = \frac{x^{\alpha-1}(1-x)^{\beta-1}}{B(\alpha,\beta)},$$

where B(α, β) denotes the value of the beta function for given values of α and β, and is a normalising constant to ensure that the beta density function integrates to 1.

Supplementary Figure 1

Supplementary Figure 1 Illustration of the beta distribution with varying shape parameters.
α > 1, β > 1 | **α > 1, β > 1, β> α**: Unimodal distribution with a peak within the interval (0, 1). As α and β increase, the peak becomes sharper and more centred, if α=β .
α < 1, β < 1: U-shaped distribution with peaks at the endpoints of the interval.
α > 1, β < 1: Right-skewed distribution.
α < 1, β > 1: Left-skewed distribution.
The estimated density functions that are presented in the results section represent an approximation of how likely values are distributed across the MIC scale for each antibiotic and specific stratum per year.

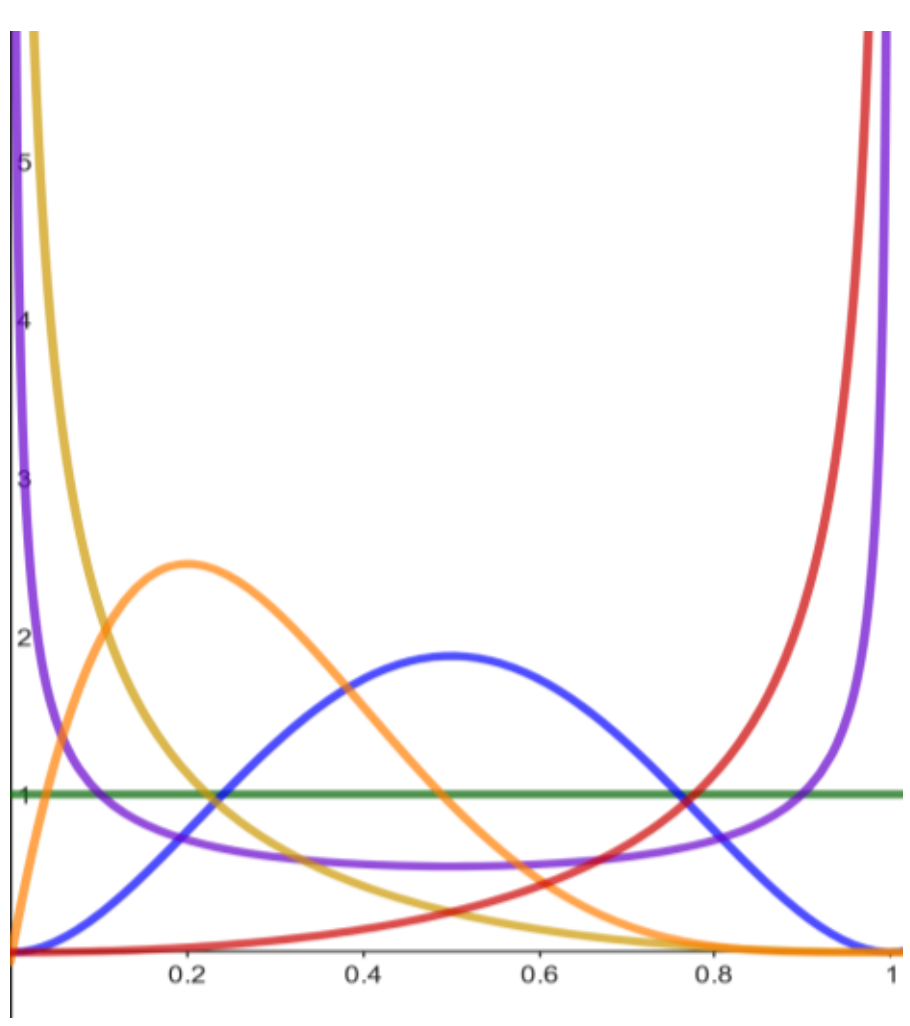

Supplementary Figure 2

Supplementary Figure 2 Empiric MIC metrics of *Neisseria gonorrhoeae* isolates submitted to the German Go-Surv-AMR sentinel surveillance system across years, Germany, 2014-2023. Epidemiological Cut-Off Value (ECOFF) for azithromycin and clinical breakpoints for ceftriaxone and ciprofloxacin are shown according to EUCAST. The horizontal black lines indicate the median values of each year.

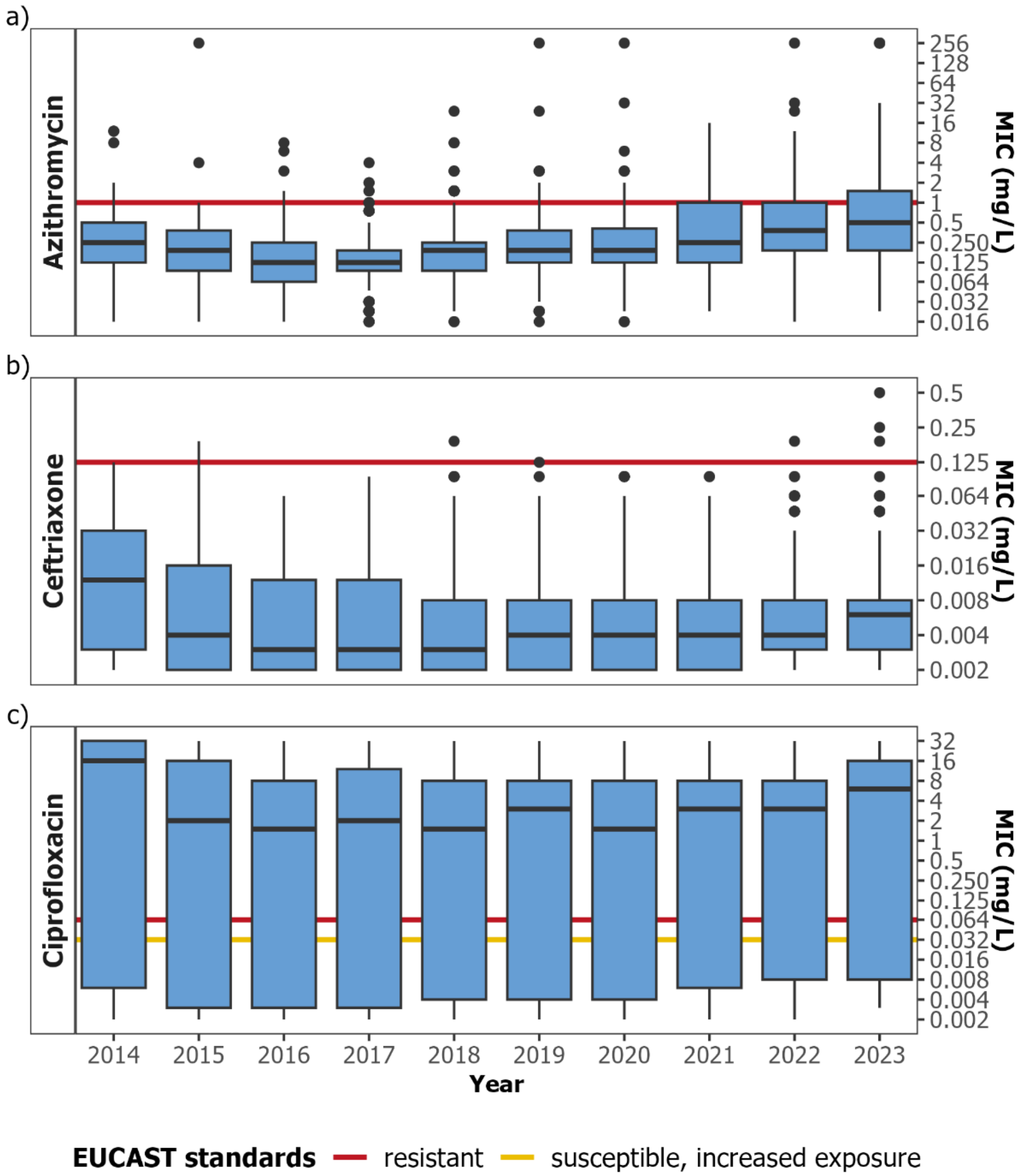

Supplementary Figure 3

Supplementary Figure 3. Empirical cumulative distribution functions (ECDFs) of the transformed and scaled MIC of *Neisseria gonorrhoeae* isolates and values derived from the fitted beta distributions for A) Azithromycin, B) Ceftriaxone and C) Ciprofloxacin, Germany 2014-2023

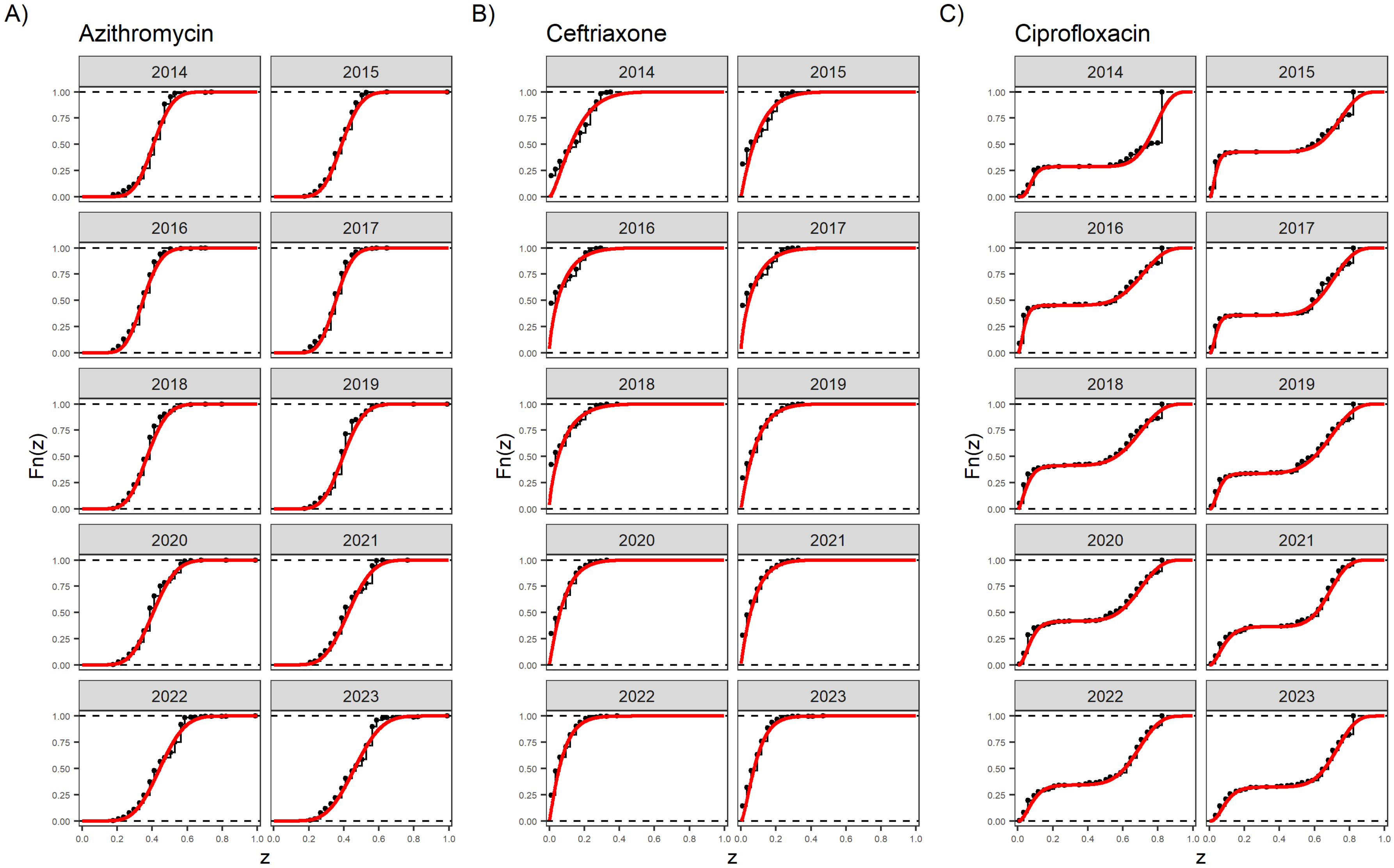

Supplementary Table 1

Supplementary Table 1 Shape parameters and characteristics of beta distributions of MIC values for azithromycin (n = 5421) and ceftriaxone (n = 5419) in *Neisseria gonorrhoeae* isolates submitted to the German Go-Surv-AMR sentinel surveillance system across years, Germany, 2014-2023

Antibiotic	Year	N	Shape parameters		Distribution characteristics			
			alpha	beta	Mean	Median	25% quantile	75% quantile
Azithromycin								
	2014	311	13.5533	19.6261	0.2439	0.2386	0.1222	0.4754
	2015	352	12.7019	19.8689	0.1962	0.1910	0.0980	0.3818
	2016	513	12.3830	22.9761	0.1229	0.1189	0.0638	0.2284
	2017	516	14.4252	26.0670	0.1320	0.1283	0.0715	0.2364
	2018	429	11.0217	18.4597	0.1623	0.1569	0.0785	0.3238
	2019	472	11.3945	17.2169	0.2163	0.2102	0.1028	0.4414
	2020	520	9.4533	13.7274	0.2420	0.2344	0.1055	0.5367
	2021	428	9.1723	12.2742	0.3057	0.2976	0.1286	0.7066
	2022	942	8.9085	11.1330	0.3726	0.3644	0.1518	0.8930
	2023	938	7.9256	9.2289	0.4578	0.4497	0.1733	1.1867
Ceftriaxone								
	2014	311	1.5598	9.2404	0.0109	0.0084	0.0043	0.0212
	2015	352	1.0411	9.7657	0.0062	0.0047	0.0029	0.0100
	2016	513	0.7181	9.0387	0.0048	0.0035	0.0024	0.0069
	2017	516	0.7113	8.9464	0.0048	0.0034	0.0024	0.0069
	2018	429	0.7209	8.3860	0.0051	0.0036	0.0024	0.0076
	2019	472	1.0251	11.0923	0.0054	0.0042	0.0027	0.0082
	2020	518	1.0703	12.1060	0.0052	0.0041	0.0027	0.0077
	2021	428	1.0015	12.3462	0.0048	0.0038	0.0026	0.0070
	2022	942	1.1362	14.2215	0.0048	0.0039	0.0027	0.0068
	2023	938	1.6418	16.6486	0.0057	0.0048	0.0032	0.0086

Supplementary Table 2

Supplementary Table 2 Shape parameters and characteristics of beta distributions of MIC values for azithromycin in *Neisseria gonorrhoeae* isolates submitted to the German Go-Surv-AMR sentinel surveillance system across years, stratified by sex, Germany, 2014-2023, n = 5295

Azithromycin								
			Shape parameters		Distribution characteristics			
Level	Year	N	alpha	beta	Mean	Median	25% quantile	75% quantile
male								
	2014	281	13.515495	19.374421	0.251040	0.245704	0.125385	0.491216
	2015	316	12.843874	20.119262	0.195444	0.190323	0.098042	0.378715
	2016	460	13.244560	24.261516	0.127217	0.123333	0.067304	0.232610
	2017	452	15.042723	26.912084	0.135581	0.132013	0.074218	0.240691
	2018	387	10.735836	17.867882	0.165166	0.159545	0.078908	0.333202
	2019	404	12.010729	18.227081	0.213629	0.207940	0.103717	0.427532
	2020	458	9.742677	13.829150	0.258188	0.250732	0.113285	0.570447
	2021	358	9.031677	12.030429	0.309786	0.301537	0.129217	0.721818
	2022	782	9.206954	11.384117	0.384268	0.376466	0.158564	0.911306
	2023	806	7.609258	8.822432	0.463552	0.455269	0.171769	1.227609
female								
	2014	29	14.793155	23.864072	0.180066	0.175791	0.095481	0.330951
	2015	34	11.077286	17.466333	0.191889	0.186004	0.091327	0.390025
	2016	52	8.381478	17.698536	0.087571	0.082932	0.041164	0.175834
	2017	61	10.762375	20.907772	0.108792	0.104515	0.054567	0.207815
	2018	33	13.721460	25.003165	0.129030	0.125244	0.068951	0.233875
	2019	60	8.056556	11.760640	0.238415	0.229597	0.096960	0.563415
	2020	53	11.579373	21.799989	0.118231	0.114010	0.060258	0.223142
	2021	46	9.798552	14.151046	0.245784	0.238467	0.108629	0.538574
	2022	118	8.556138	11.905151	0.273320	0.264726	0.112630	0.641289
	2023	105	9.976565	12.607960	0.360667	0.353323	0.155043	0.820928

Supplementary Table 3

Supplementary Table 3 Shape parameters and characteristics of beta distributions of MIC values for azithromycin in *Neisseria gonorrhoeae* isolates submitted to the German Go-Surv-AMR sentinel surveillance system across years, stratified by age group, Germany, 2014-2023, n = 5352

Azithromycin								
			Shape parameters		Distribution characteristics			
Level	Year	N	alpha	beta	Mean	Median	25% quantile	75% quantile
under 25								
	2014	75	9.427762	13.487192	0.252498	0.244837	0.109489	0.563609
	2015	74	14.458734	24.748273	0.152920	0.148922	0.081720	0.278142
	2016	105	13.005765	25.576225	0.105352	0.101889	0.056545	0.189383
	2017	105	12.319319	23.043170	0.120293	0.116276	0.062502	0.223260
	2018	69	7.904664	12.936188	0.173033	0.165215	0.072426	0.393709
	2019	98	11.316656	17.846429	0.191809	0.186049	0.092037	0.386961
	2020	137	7.514243	11.183838	0.225672	0.216393	0.089367	0.545262
	2021	84	8.577229	12.247160	0.253868	0.245433	0.105533	0.589302
	2022	176	8.732749	10.895405	0.374348	0.366040	0.151113	0.905696
	2023	187	8.672641	10.715757	0.385071	0.376792	0.154555	0.937690
25-34								
	2014	103	13.044800	19.991861	0.207804	0.202625	0.104220	0.403315
	2015	119	9.217380	13.889411	0.217928	0.210460	0.095027	0.481666
	2016	169	10.634820	19.251945	0.131330	0.126397	0.064144	0.258135
	2017	184	15.974486	28.340472	0.138694	0.135284	0.077160	0.242724
	2018	141	10.576813	17.287728	0.173629	0.167768	0.082036	0.354298
	2019	158	12.693586	19.300729	0.212466	0.207095	0.105310	0.417095
	2020	179	8.451263	12.265627	0.242364	0.233915	0.100572	0.562598
	2021	167	8.836894	11.503469	0.331027	0.322612	0.135871	0.784886
	2022	332	9.136273	11.431625	0.371241	0.363282	0.153111	0.879778
	2023	297	8.253459	9.470637	0.477826	0.470466	0.183908	1.221416
35-44								
	2014	65	13.293870	18.387689	0.278011	0.272468	0.136861	0.552723
	2015	76	18.294756	28.304299	0.202356	0.198703	0.113479	0.353845

Azithromycin								
			Shape parameters		Distribution characteristics			
Level	Year	N	alpha	beta	Mean	Median	25% quantile	75% quantile
	2016	111	12.116755	21.043325	0.146965	0.142311	0.074306	0.280847
	2017	96	14.580410	25.196763	0.148967	0.145063	0.080021	0.269531
	2018	106	11.163380	19.793937	0.138905	0.134030	0.068623	0.270653
	2019	95	8.814262	12.898323	0.236761	0.228719	0.100363	0.538498
	2020	94	12.439273	17.526810	0.263689	0.257824	0.127248	0.533477
	2021	74	10.252299	13.152070	0.345340	0.338130	0.150754	0.773600
	2022	188	8.510197	10.357008	0.402417	0.394150	0.159469	0.993555
	2023	206	6.962092	7.838982	0.505064	0.496996	0.177371	1.414262
45+								
	2014	64	30.448244	43.412206	0.254934	0.252557	0.160927	0.399788
	2015	81	15.638185	24.142130	0.203584	0.199300	0.108732	0.372587
	2016	128	16.914926	32.783645	0.109464	0.106722	0.063325	0.184124
	2017	127	14.038553	26.252646	0.120371	0.116841	0.065258	0.215055
	2018	106	15.278079	25.622696	0.161725	0.157817	0.087495	0.291189
	2019	120	12.703679	18.793339	0.229572	0.224048	0.113021	0.454347
	2020	106	12.917134	18.724015	0.243215	0.237681	0.119847	0.481596
	2021	91	9.658485	13.442408	0.273121	0.265510	0.118744	0.609677
	2022	221	9.298425	11.930633	0.345153	0.337199	0.144411	0.804872
	2023	238	7.825070	9.256320	0.437188	0.428673	0.165127	1.133886

Supplementary Table 4

Supplementary Table 4 Shape parameters and characteristics of beta distributions of MIC values for azithromycin in *Neisseria gonorrhoeae* isolates submitted to the German Go-Surv-AMR sentinel surveillance system across years, stratified by settlement structure, Germany, 2014-2023, n = 5420

Azithromycin								
			Shape parameters		Distribution characteristics			
Level	Year	N	alpha	beta	Mean	Median	25% quantile	75% quantile
Countryside								
	2014	92	12.978544	19.047946	0.234799	0.229351	0.116258	0.462469
	2015	111	12.081247	19.574242	0.177904	0.172704	0.088112	0.348003
	2016	182	10.959620	21.137472	0.110891	0.106632	0.055852	0.211153
	2017	136	12.806686	24.930651	0.108201	0.104616	0.057615	0.195998
	2018	97	10.719116	18.690408	0.145376	0.140154	0.070394	0.288747
	2019	99	14.804534	23.633728	0.185386	0.181052	0.098070	0.341672
	2020	139	8.270459	12.420859	0.219997	0.211645	0.091364	0.508534
	2021	121	9.051584	12.512889	0.278456	0.270308	0.117468	0.639679
	2022	325	9.151175	11.379510	0.377985	0.370087	0.155764	0.897030
	2023	316	8.686813	10.088310	0.461336	0.454054	0.182503	1.146827
Metropolitan city								
	2014	130	12.851161	18.672606	0.241582	0.236031	0.118892	0.478852
	2015	131	11.675461	17.540368	0.219793	0.213876	0.105301	0.445620
	2016	179	15.087552	26.768683	0.138670	0.135061	0.075814	0.246561
	2017	227	15.820048	27.204526	0.150987	0.147360	0.083096	0.267277
	2018	204	11.731945	18.773024	0.184170	0.178721	0.089933	0.365264
	2019	231	9.702178	14.545585	0.221086	0.213952	0.098363	0.479938
	2020	228	9.936312	14.895708	0.221123	0.214156	0.099353	0.475698
	2021	164	8.593144	11.671925	0.292872	0.284230	0.120087	0.692055
	2022	327	7.995876	10.200989	0.350903	0.341670	0.136657	0.876147
	2023	433	7.425580	8.801602	0.434606	0.425585	0.159943	1.155321

Azithromycin								
			Shape parameters		Distribution characteristics			
Level	Year	N	alpha	beta	Mean	Median	25% quantile	75% quantile
Medium-sized city								
	2014	88	15.016423	21.396798	0.255381	0.250559	0.132095	0.483758
	2015	110	15.263555	24.188086	0.189222	0.184976	0.100911	0.346290
	2016	152	11.798047	22.044910	0.120632	0.116430	0.061740	0.226934
	2017	153	14.995227	27.332952	0.128919	0.125455	0.070849	0.227827
	2018	128	10.486302	18.334524	0.144298	0.138985	0.069356	0.288457
	2019	142	13.139780	19.357912	0.232288	0.226926	0.115635	0.455125
	2020	153	10.355107	13.918638	0.301822	0.294639	0.133884	0.663250
	2021	143	10.049217	12.863726	0.347507	0.340158	0.150330	0.785328
	2022	290	9.813473	12.049208	0.392190	0.384938	0.166216	0.907300
	2023	189	7.948030	8.926341	0.508846	0.501882	0.191265	1.334353

Supplementary Table 5

Supplementary Table 5 Shape parameters and characteristics of beta distributions of MIC values for azithromycin in *Neisseria gonorrhoeae* isolates submitted to the German Go-Surv-AMR sentinel surveillance system across years, stratified by specimen in the male subsample, Germany, 2014-2023, n = 4356

Azithromycin								
			Shape parameters		Distribution characteristics			
Level	Year	N	alpha	beta	Mean	Median	25% quantile	75% quantile
urethral								
	2014	263	12.903458	18.663834	0.244712	0.239163	0.120473	0.485048
	2015	301	12.528585	19.809827	0.190402	0.185216	0.094908	0.370878
	2016	417	13.235054	24.635112	0.121877	0.118102	0.064755	0.221785
	2017	395	15.342905	27.584947	0.133784	0.130316	0.073775	0.235859

Azithromycin								
			Shape parameters		Distribution characteristics			
Level	Year	N	alpha	beta	Mean	Median	25% quantile	75% quantile
	2018	337	10.899023	18.467571	0.157218	0.151835	0.075945	0.313606
	2019	337	11.596622	18.106822	0.197221	0.191527	0.095242	0.395837
	2020	412	10.628214	15.351298	0.245691	0.238945	0.112277	0.521962
	2021	304	8.833734	12.137236	0.283382	0.275010	0.118047	0.659079
	2022	607	8.908525	11.487588	0.340200	0.331878	0.139797	0.806538
	2023	628	7.627384	9.199952	0.413040	0.403942	0.154795	1.076711
extragenital								
	2014	9	121.494396	165.177479	0.292090	0.291481	0.231442	0.367794
	2015	9	69.913029	82.804325	0.435590	0.434645	0.315915	0.599187
	2016	20	10.584083	16.672701	0.192403	0.186236	0.089950	0.397533
	2017	29	18.823242	30.573253	0.176687	0.173358	0.100994	0.302851
	2018	19	9.094043	13.099847	0.247589	0.239688	0.105908	0.559287
	2019	32	22.309847	28.697307	0.342654	0.339347	0.196220	0.592153
	2020	28	4.090653	4.894490	0.422846	0.405986	0.108931	1.575501
	2021	29	17.494891	16.992954	0.779471	0.780777	0.396084	1.536706
	2022	82	22.280440	22.104232	0.732441	0.732700	0.403218	1.330978
	2023	98	11.915825	11.953344	0.708959	0.708773	0.313358	1.603541

Supplementary Table 6

Supplementary Table 6 Shape parameters and characteristics of beta distributions of MIC values for ceftriaxone in *Neisseria gonorrhoeae* isolates submitted to the German Go-Surv-AMR sentinel surveillance system across years, stratified by sex, Germany, 2014-2023, n = 5293

Ceftriaxone								
			Shape parameters		Distribution characteristics			
Level	Year	N	alpha	beta	Mean	Median	25% quantile	75% quantile
male								
	2014	281	1.531389	9.057750	0.010955	0.008416	0.004303	0.021375
	2015	316	1.018794	9.748742	0.006085	0.004572	0.002851	0.009753
	2016	460	0.692527	8.851635	0.004695	0.003394	0.002370	0.006754
	2017	452	0.697712	8.889839	0.004706	0.003407	0.002375	0.006779
	2018	387	0.714261	8.433587	0.005010	0.003578	0.002423	0.007437
	2019	404	1.034637	11.219477	0.005398	0.004177	0.002749	0.008183
	2020	456	1.089983	12.174350	0.005257	0.004141	0.002764	0.007845
	2021	358	1.012609	12.881582	0.004712	0.003741	0.002610	0.006731
	2022	782	1.115353	14.503438	0.004632	0.003765	0.002658	0.006530
	2023	806	1.633778	16.442706	0.005789	0.004860	0.003233	0.008647
female								
	2014	29	1.940972	11.324193	0.011177	0.009060	0.004829	0.020855
	2015	34	1.162985	10.001740	0.006808	0.005186	0.003110	0.011329
	2016	52	0.942613	10.805848	0.005138	0.003931	0.002633	0.007660
	2017	61	0.845309	9.352223	0.005301	0.003910	0.002576	0.008060
	2018	33	0.606036	6.922058	0.005154	0.003460	0.002335	0.007738
	2019	60	0.956768	10.001533	0.005584	0.004204	0.002715	0.008643
	2020	53	0.862493	10.560615	0.004860	0.003688	0.002527	0.007089
	2021	46	0.960509	10.095873	0.005555	0.004192	0.002714	0.008576
	2022	118	1.417528	13.579306	0.006078	0.004936	0.003170	0.009412
	2023	105	1.783717	18.114829	0.005739	0.004893	0.003299	0.008455

Supplementary Table 7

Supplementary Table 7 Shape parameters and characteristics of beta distributions of MIC values for ceftriaxone in *Neisseria gonorrhoeae* isolates submitted to the German Go-Surv-AMR sentinel surveillance system across years, stratified by settlement structure, Germany, 2014-2023, n = 5418

Ceftriaxone								
			Shape parameters		Distribution characteristics			
Level	Year	N	alpha	beta	Mean	Median	25% quantile	75% quantile
Countryside								
	2014	92	2.172208	11.417030	0.013104	0.010752	0.005553	0.025227
	2015	111	1.000086	8.979377	0.006499	0.004792	0.002898	0.010758
	2016	182	0.903478	9.462631	0.005574	0.004134	0.002666	0.008650
	2017	136	0.731687	8.704832	0.004978	0.003588	0.002435	0.007367
	2018	97	0.805617	9.673076	0.004939	0.003666	0.002493	0.007273
	2019	99	0.829143	8.989253	0.005399	0.003941	0.002574	0.008286
	2020	138	0.933091	10.367519	0.005281	0.004003	0.002648	0.007978
	2021	121	0.930075	11.079024	0.004972	0.003822	0.002596	0.007307
	2022	325	1.265650	15.925285	0.004754	0.003937	0.002770	0.006718
	2023	316	1.736699	17.403053	0.005814	0.004927	0.003296	0.008635
Metropolitan city								
	2014	130	1.211331	7.819713	0.009684	0.007062	0.003651	0.018775
	2015	131	0.909910	9.900181	0.005382	0.004034	0.002644	0.008212
	2016	179	0.590791	9.167431	0.004076	0.002973	0.002229	0.005456
	2017	227	0.649065	8.409257	0.004645	0.003309	0.002328	0.006640
	2018	204	0.654936	7.236152	0.005308	0.003619	0.002397	0.008103
	2019	231	0.988642	10.879084	0.005327	0.004089	0.002700	0.008052
	2020	227	1.054943	13.263042	0.004757	0.003802	0.002646	0.006807
	2021	164	1.149186	14.323989	0.004790	0.003890	0.002711	0.006838
	2022	327	1.047326	13.724625	0.004604	0.003701	0.002612	0.006497
	2023	433	1.675869	17.902338	0.005473	0.004648	0.003169	0.007974
Medium-sized city								
	2014	88	1.653178	9.767420	0.010973	0.008592	0.004459	0.021066
	2015	110	1.279360	10.685566	0.007033	0.005458	0.003261	0.011731

Ceftriaxone								
			Shape parameters		Distribution characteristics			
Level	Year	N	alpha	beta	Mean	Median	25% quantile	75% quantile
	2016	152	0.688574	8.776540	0.004705	0.003394	0.002367	0.006776
	2017	153	0.789575	10.002705	0.004728	0.003535	0.002452	0.006822
	2018	128	0.801663	9.918490	0.004819	0.003597	0.002474	0.007014
	2019	142	1.271591	13.377943	0.005551	0.004474	0.002951	0.008371
	2020	153	1.273099	12.681791	0.005847	0.004672	0.003016	0.009015
	2021	143	0.915529	11.480867	0.004767	0.003689	0.002552	0.006876
	2022	290	1.112471	13.175690	0.004997	0.003995	0.002730	0.007282
	2023	189	1.495926	13.820214	0.006307	0.005149	0.003278	0.009852

Supplementary Table 8

Supplementary Table 8 Shape parameters and characteristics of beta distributions of MIC values for ceftriaxone in *Neisseria gonorrhoeae* isolates submitted to the German Go-Surv-AMR sentinel surveillance system across years, stratified by age, Germany, 2014-2023, n = 5350

Ceftriaxone								
			Shape parameters		Distribution characteristics			
Level	Year	N	alpha	beta	Mean	Median	25% quantile	75% quantile
under 25								
	2014	75	1.927918	9.847743	0.013715	0.010941	0.005399	0.027669
	2015	74	1.000162	10.172159	0.005731	0.004340	0.002776	0.008950
	2016	105	0.699023	8.809572	0.004748	0.003429	0.002381	0.006868
	2017	105	0.685314	9.220035	0.004512	0.003297	0.002344	0.006368
	2018	69	0.569777	8.198681	0.004294	0.003036	0.002233	0.005878
	2019	98	0.875496	11.442981	0.004613	0.003564	0.002500	0.006566
	2020	137	0.874843	10.763545	0.004841	0.003691	0.002534	0.007044
	2021	84	1.042443	11.837019	0.005181	0.004052	0.002716	0.007706
	2022	176	1.077572	12.910154	0.004949	0.003938	0.002697	0.007196
	2023	187	1.503014	16.444286	0.005355	0.004480	0.003047	0.007826

Ceftriaxone								
			Shape parameters		Distribution characteristics			
Level	Year	N	alpha	beta	Mean	Median	25% quantile	75% quantile
25-34								
	2014	103	1.563834	9.623421	0.010350	0.008040	0.004223	0.019639
	2015	119	0.997887	8.891139	0.006552	0.004820	0.002904	0.010890
	2016	169	0.697844	8.341065	0.004958	0.003529	0.002403	0.007324
	2017	184	0.644644	8.357725	0.004642	0.003301	0.002324	0.006633
	2018	141	0.693312	8.394018	0.004905	0.003496	0.002393	0.007208
	2019	158	1.090842	10.706837	0.005933	0.004561	0.002889	0.009344
	2020	179	1.195682	13.956187	0.005059	0.004096	0.002796	0.007373
	2021	167	0.934224	10.905065	0.005059	0.003876	0.002613	0.007491
	2022	332	1.106127	15.154862	0.004451	0.003644	0.002615	0.006170
	2023	297	1.717682	16.686991	0.005993	0.005051	0.003335	0.009018
35-44								
	2014	65	1.342680	8.120523	0.010609	0.007887	0.003987	0.020996
	2015	76	1.225859	11.245276	0.006354	0.004962	0.003082	0.010195
	2016	111	0.752404	9.192067	0.004869	0.003562	0.002440	0.007129
	2017	96	0.824811	9.163589	0.005282	0.003872	0.002555	0.008023
	2018	106	0.675879	8.118924	0.004938	0.003485	0.002381	0.007277
	2019	95	0.936466	9.811534	0.005572	0.004174	0.002696	0.008628
	2020	92	1.159874	12.293740	0.005512	0.004361	0.002865	0.008358
	2021	74	1.007673	15.450273	0.004109	0.003368	0.002493	0.005526
	2022	188	1.256744	14.546412	0.005095	0.004160	0.002842	0.007419
	2023	206	1.380772	13.620328	0.005904	0.004790	0.003104	0.009059
45+								
	2014	64	1.348102	8.940556	0.009337	0.007058	0.003769	0.017466
	2015	81	0.991798	9.387368	0.006153	0.004578	0.002836	0.009935
	2016	128	0.731910	10.171035	0.004404	0.003301	0.002367	0.006150
	2017	127	0.736495	9.172323	0.004793	0.003502	0.002417	0.006966
	2018	106	0.865141	8.591383	0.005865	0.004242	0.002671	0.009347

Ceftriaxone								
			Shape parameters		Distribution characteristics			
Level	Year	N	alpha	beta	Mean	Median	25% quantile	75% quantile
	2019	120	1.183041	12.951707	0.005352	0.004277	0.002849	0.007997
	2020	106	1.123714	11.271727	0.005808	0.004516	0.002893	0.009037
	2021	91	1.119661	13.910741	0.004803	0.003877	0.002696	0.006876
	2022	221	1.256902	15.506936	0.004830	0.003983	0.002782	0.006875
	2023	238	1.979647	20.267477	0.005695	0.004935	0.003379	0.008263

Supplementary Table 9

Supplementary Table 9 Shape parameters and characteristics of beta distributions of MIC values for ciprofloxacin in *Neisseria gonorrhoeae* isolates submitted to the German Go-Surv-AMR sentinel surveillance system across years, Germany, 2014-2023, n = 5421

Antibiotic	Year	N	First beta distribution							Second beta distribution					
			Shape parameters			Distribution characteristics				Shape parameters		Distribution characteristics			
			alpha 1	beta 1	p1	Mean	Median	25% quantile	75% quantile	alpha 2	beta 2	Mean	Median	25% quantile	75% quantile
Ciprofloxacin															
	2014	311	5.19	57.91	0.29	0.0053	0.0050	0.0039	0.0067	20.05	6.04	16.80	18.22	9.15	33.62
	2015	352	2.60	63.28	0.43	0.0032	0.0030	0.0026	0.0037	15.21	5.72	10.31	11.24	4.99	23.30
	2016	513	2.29	55.26	0.45	0.0032	0.0030	0.0026	0.0037	11.88	5.28	6.86	7.50	2.97	17.39
	2017	516	2.79	62.48	0.36	0.0033	0.0031	0.0027	0.0039	13.77	6.17	6.72	7.26	3.08	15.91
	2018	429	1.84	29.95	0.41	0.0040	0.0036	0.0028	0.0051	11.50	5.44	5.86	6.37	2.49	15.07
	2019	472	2.62	43.38	0.34	0.0039	0.0036	0.0029	0.0048	10.89	5.21	5.68	6.20	2.35	15.01
	2020	520	2.61	31.33	0.42	0.0049	0.0045	0.0033	0.0066	13.80	6.37	6.25	6.72	2.86	14.77
	2021	428	2.21	22.19	0.36	0.0058	0.0051	0.0035	0.0084	19.45	9.27	5.75	6.03	2.95	11.79
	2022	942	2.51	25.75	0.34	0.0057	0.0051	0.0036	0.0080	14.10	6.69	5.82	6.23	2.68	13.61
	2023	938	2.99	27.54	0.32	0.0063	0.0057	0.0040	0.0090	16.29	6.54	8.82	9.50	4.34	19.40

Supplementary Table 10

Supplementary Table 10 Shape parameters and characteristics of beta distributions of MIC values for ciprofloxacin in *Neisseria gonorrhoeae* isolates submitted to the German Go-Surv-AMR sentinel surveillance system across years, stratified by sex, Germany, 2014-2023, n= 5295

Ciprofloxacin															
			First beta distribution							Second beta distribution					
			Shape parameters			Distribution characteristics				Shape parameters		Distribution characteristics			
Level	Year	N	alpha 1	beta 1	p1	Mean	Median	25% quantile	75% quantile	alpha 2	beta 2	Mean	Median	25% quantile	75% quantile
male															
	2014	281	4.07	42.53	0.29	0.00558	0.00521	0.00391	0.00739	23.35	6.87	17.66	18.96	10.05	33.45
	2015	316	2.56	62.96	0.44	0.00317	0.00300	0.00257	0.00368	14.87	5.67	9.99	10.89	4.79	22.81
	2016	460	2.61	63.12	0.46	0.00319	0.00302	0.00259	0.00371	12.32	5.50	6.79	7.39	2.98	16.91
	2017	452	2.81	61.82	0.37	0.00334	0.00316	0.00268	0.00392	14.81	6.53	7.01	7.54	3.30	16.07
	2018	387	2.37	42.48	0.40	0.00372	0.00345	0.00279	0.00458	9.80	4.83	5.29	5.81	2.09	14.75
	2019	404	2.63	43.98	0.33	0.00388	0.00361	0.00290	0.00481	10.32	5.09	5.27	5.75	2.13	14.29
	2020	458	2.54	29.78	0.40	0.00504	0.00456	0.00335	0.00682	13.79	6.37	6.23	6.70	2.85	14.73
	2021	358	2.22	23.03	0.34	0.00563	0.00496	0.00346	0.00802	20.43	9.90	5.50	5.76	2.87	11.09
	2022	782	2.52	25.97	0.34	0.00565	0.00505	0.00357	0.00794	13.88	6.60	5.78	6.20	2.64	13.62
	2023	806	2.98	27.22	0.31	0.00638	0.00575	0.00400	0.00913	16.51	6.65	8.75	9.41	4.32	19.15
female															
	2014	29	6.09	85.26	0.24	0.00438	0.00422	0.00351	0.00526	15.78	5.07	14.66	16.17	7.36	32.36
	2015	34	3.49	75.20	0.35	0.00337	0.00322	0.00276	0.00392	17.42	6.12	12.04	13.06	6.15	25.67
	2016	52	0.83	15.13	0.38	0.00370	0.00301	0.00233	0.00476	13.88	5.68	8.41	9.15	3.90	19.82
	2017	61	1.42	27.15	0.34	0.00359	0.00318	0.00253	0.00449	12.86	5.90	6.35	6.87	2.83	15.49
	2018	33	1.26	19.05	0.61	0.00415	0.00353	0.00262	0.00554	21.02	7.95	10.15	10.79	5.44	20.21
	2019	60	2.82	46.29	0.37	0.00393	0.00366	0.00295	0.00486	18.87	7.13	10.17	10.90	5.28	21.09
	2020	53	5.37	87.86	0.55	0.00394	0.00379	0.00320	0.00465	12.59	5.58	6.90	7.51	3.06	17.02
	2021	46	2.26	22.88	0.52	0.00576	0.00507	0.00352	0.00826	20.78	8.63	8.11	8.58	4.30	16.26
	2022	118	3.00	30.26	0.31	0.00578	0.00525	0.00377	0.00801	14.20	6.78	5.72	6.12	2.64	13.33
	2023	105	3.08	32.38	0.36	0.00556	0.00507	0.00370	0.00758	15.77	6.45	8.43	9.08	4.09	18.80

Supplementary Table 11

Supplementary Table 11 Shape parameters and characteristics of beta distributions of MIC values for ciprofloxacin in *Neisseria gonorrhoeae* isolates submitted to the German Go-Surv-AMR sentinel surveillance system across years, stratified by settlement structure, Germany, 2014-2023, n = 5420

Ciprofloxacin															
			First beta distribution							Second beta distribution					
			Shape parameters			Distribution characteristics				Shape parameters		Distribution characteristics			
Level	Year	N	alpha 1	beta 1	p1	Mean	Median	25% quantile	75% quantile	alpha 2	beta 2	Mean	Median	25% quantile	75% quantile
Countryside															
	2014	92	7.45	81.70	0.30	0.00535	0.00515	0.00417	0.00659	34.61	9.24	21.46	22.61	13.58	35.88
	2015	111	1.76	39.12	0.41	0.00332	0.00305	0.00252	0.00399	16.02	5.60	12.20	13.33	6.08	26.90
	2016	182	2.24	59.19	0.34	0.00307	0.00290	0.00250	0.00355	14.79	6.37	7.42	8.00	3.50	17.02
	2017	136	2.84	76.87	0.29	0.00304	0.00291	0.00254	0.00347	12.90	6.11	5.83	6.29	2.59	14.20
	2018	97	1.80	26.10	0.37	0.00427	0.00379	0.00286	0.00563	10.72	5.31	5.22	5.68	2.14	13.88
	2019	99	3.75	70.21	0.37	0.00363	0.00346	0.00292	0.00430	12.35	5.35	7.30	7.98	3.22	18.17
	2020	139	3.92	51.78	0.48	0.00458	0.00431	0.00341	0.00576	11.37	5.41	5.77	6.28	2.44	14.94
	2021	121	2.44	30.08	0.37	0.00483	0.00437	0.00324	0.00647	18.34	8.63	5.94	6.26	2.99	12.47
	2022	325	2.25	21.32	0.36	0.00615	0.00538	0.00365	0.00903	15.70	7.10	6.58	7.03	3.16	14.70
	2023	316	3.33	32.15	0.31	0.00603	0.00552	0.00396	0.00836	17.30	6.89	8.98	9.64	4.51	19.28
Metropolitan city															
	2014	130	4.90	54.87	0.31	0.00524	0.00497	0.00387	0.00670	14.06	4.65	13.77	15.32	6.63	32.00
	2015	131	2.49	57.41	0.47	0.00326	0.00308	0.00261	0.00383	16.11	6.80	7.81	8.39	3.81	17.28
	2016	179	3.32	82.75	0.57	0.00315	0.00302	0.00263	0.00360	9.40	4.22	6.68	7.47	2.62	19.12
	2017	227	3.42	70.81	0.41	0.00344	0.00328	0.00279	0.00403	14.30	6.01	7.88	8.54	3.69	18.35
	2018	204	2.65	50.69	0.44	0.00359	0.00336	0.00277	0.00432	8.83	4.21	5.73	6.39	2.17	16.96
	2019	231	1.87	28.83	0.34	0.00409	0.00367	0.00282	0.00530	12.46	5.94	5.76	6.22	2.52	14.25
	2020	228	2.16	27.73	0.40	0.00468	0.00419	0.00311	0.00627	14.60	6.85	5.97	6.39	2.78	13.76
	2021	164	2.38	23.22	0.34	0.00597	0.00528	0.00364	0.00862	22.10	11.01	5.14	5.35	2.74	10.05
	2022	327	2.61	26.81	0.36	0.00568	0.00509	0.00361	0.00796	15.18	7.11	6.02	6.42	2.84	13.64
	2023	433	2.53	21.32	0.32	0.00697	0.00613	0.00404	0.01050	18.12	7.30	8.74	9.34	4.45	18.42

Ciprofloxacin																
			First beta distribution							Second beta distribution						
			Shape parameters			Distribution characteristics				Shape parameters		Distribution characteristics				
Level	Year	N	alpha 1	beta 1	p1	Mean	Median	25% quantile	75% quantile	alpha 2	beta 2	Mean	Median	25% quantile	75% quantile
Medium-sized city																
	2014	88	3.83	43.85	0.23	0.00514	0.00480	0.00367	0.00670	26.82	7.88	17.72	18.85	10.45	32.10
	2015	110	2.84	70.73	0.39	0.00315	0.00300	0.00260	0.00363	21.99	7.45	13.06	13.95	7.18	25.48
	2016	152	1.35	25.10	0.47	0.00365	0.00321	0.00252	0.00460	15.95	6.91	7.32	7.85	3.55	16.27
	2017	153	1.77	35.49	0.35	0.00349	0.00318	0.00258	0.00428	17.92	8.06	6.65	7.05	3.34	14.10
	2018	128	1.60	25.86	0.39	0.00397	0.00351	0.00270	0.00514	14.16	6.81	5.62	6.01	2.58	13.11
	2019	142	3.59	55.95	0.31	0.00407	0.00384	0.00312	0.00499	9.63	4.82	5.06	5.55	1.98	14.25
	2020	153	3.15	33.05	0.38	0.00557	0.00510	0.00372	0.00759	12.73	5.73	6.64	7.20	2.95	16.28
	2021	143	2.12	18.95	0.39	0.00654	0.00564	0.00371	0.00988	18.37	8.41	6.38	6.74	3.22	13.38
	2022	290	2.68	29.82	0.31	0.00527	0.00477	0.00347	0.00719	12.63	6.36	5.00	5.36	2.19	12.26
	2023	189	3.48	34.98	0.35	0.00579	0.00533	0.00389	0.00789	13.20	5.21	9.16	10.06	4.19	22.08
